# Nontuberculous Mycobacterium Infection and Tumor Necrosis Factor-α Antagonists

**DOI:** 10.3201/eid1510.090110

**Published:** 2009-10

**Authors:** Reinout M. Swart, Jakko van Ingen, Dick van Soolingen, Rob Slingerland, Willem D.H. Hendriks, Jan G. den Hollander

**Affiliations:** Maasstad Ziekenhuis Rotterdam, Rotterdam, the Netherlands (R.M. Swart, R. Slingerland, W.D.H. Hendriks, J.G. den Hollander); National Institute for Public Health and the Environment, Bilthoven, the Netherlands (J. van Ingen, D. van Soolingen).

**Keywords:** Tuberculosis and other mycobacteria, nontuberculous mycobacteria, pneumonia, rheumatoid arthritis, tumor necrosis factor-α antagonists, adalimumab, the Netherlands, bacteria, letter

**To the Editor**: *Mycobacterium haemophilum* is an aerobic, slow-growing microorganism with optimal growth at 30°C to 32°C. It has a unique requirement for ferric iron–containing compounds (*1*), from which it acquired its name (i.e., *haemophilum*). Infections with *M*. *haemophilum* are rare, but cervicofacial lymphadenitis caused by *M*. *haemophilum* has been described in children (*2*). Besides cervicofacial lymphadenitis, extrapulmonary signs of *M*. *haemophilum* disease include subcutaneous noduli, arthritis, and osteomyelitis, which generally affect immunocompromised patients (*3*). Recently, 2 cases of cutaneous *M*. *haemophilum* infections after alemtuzumab treatment were reported (*4*). A small number of pulmonary *M*. *haemophilum* infections associated with AIDS or solid organ or bone marrow transplantation have been described (*1*). We report pulmonary *M*. *haemophilum* infection in a woman who had been immunosuppressed by tumor necrosis factor-α antagonist (TNF-αA) (adalimumab) treatment for rheumatoid arthritis.

A 72-year-old woman with a history of rheumatoid arthritis and obstructive sleep apnea syndrome had signs and symptoms of fatigue, mild fever episodes, and a nonproductive cough 9 months after treatment for rheumatoid arthritis had begun with methotrexate (MTX) and TNF-αA. Physical examination was unremarkable except for a body temperature of 38.9°C. Laboratory testing showed an increased erythrocyte sedimentation rate (ESR) (77 mm/h), an increased C-reactive protein (CRP) level (60 mg/L), a normal leukocyte count (8,500 cells/μL), and relative monocytosis (12%). HIV serologic testing results were negative. Chest radiograph showed an infiltrate in the right upper lobe. Chest computed tomography confirmed this finding and showed lymphadenopathy in the right hilus and mediastinum.

Notably, the tuberculin skin test result was negative at screening before she began the TNF-αA treatment, but was now positive (20 mm), suggesting mycobacterial infection. Auramine and Ziehl-Neelsen staining of sputum and bronchoalveolar liquids showed no acid-fast bacilli, and *M. tuberculosis* infection was not confirmed by PCR or culture. Eventually, a mediastinal lymph node biopsy was taken by endoscopic ultrasound guidance. Granulomatous inflammation and acid-fast bacilli were seen by microscopy. Corresponding cultures yielded a strain identified as *M*. *haemophilum* at the Netherlands National Institute for Public Health and the Environment (RIVM) by using the Inno-LiPA Mycobacteria v2 reverse line blot assay (Innogenetics, Ghent, Belgium). Strain identity was confirmed by sequencing of the complete 16S rDNA gene, which was identical to that of *M*. *haemophilum* available in the GenBank sequence database (National Center for Biotechnology Information; www.ncbi.nlm.nih.gov; accession no. X88923).

The RIVM performed drug susceptibility testing by using a modified agar dilution method (*5*). Middlebrook 7H10 media were enriched with 10% sheep blood hemolyzed by 1:1 dilution with water and subsequent freezing–thawing. Historic drug susceptibility data was reviewed (Table). Initially, adalimumab was discontinued, and our patient was treated with isoniazid, ethambutol, rifampin, and pyrazinimide because *M*. *tuberculosis* infection was suspected. After identification of *M*. *haemophilum*, our patient was treated with rifampin and azithromycin. A total treatment duration of 10 months resulted in complete resolution of the pulmonary infiltrate and normalization of ESR and CRP concentration. During the follow- up period of >12 months, the patient remained asymptomatic. Her rheumatoid arthritis was treated with MTX monotherapy.

**Table:** Antimicrobial drug susceptibility test results for *Mycobacterium haemophilum* isolate from rheumatoid arthritis patient and other *M. haemophilum* isolates*

Antimicrobial drug	Case report			RIVM historic data (n = 49)	
	Classification	MIC, mg/L		% Susceptible	% Resistant
Isoniazid	Resistant	10		0	100
Rifampin	Susceptible	0.2		4	96
Ethambutol	Resistant	20		0	100
Streptomycin	Susceptible	<1.0		35	65
Cycloserine	Susceptible	50		78	22
Prothionamide	Susceptible	<1.0		61	39
Amikacin	Resistant	10		29	71
Ciprofloxacin	Resistant	4.0		88	12
Clofazimine	Susceptible	<0.5		92	8
Clarithromycin	Susceptible	<2.0		94	6
Rifabutin	Susceptible	<0.2		96	4

*All isolates submitted to the Mycobacteria Reference Laboratory, National Institute for Public Health and the Environment (RIVM), the Netherlands, January 2000–January 2007. Before January 2004, strains tested were identified by 16S rDNA gene sequencing; after January 2004, strains were identified by the Inno-LiPA assay (Innogenetics, Ghent, Belgium).

This case illustrates the risk for infectious diseases during TNF-αA treatment. Rheumatoid arthritis can be treated effectively with MTX and TNFαA (*6*). Side effects of concern are infectious diseases, which prompt the need for screening for latent mycobacterial infection before commencing treatment (*7*). Despite screening, mycobacterial infections have been diagnosed after prolonged treatment in various patients (*8*). This case shows that not only *M. tuberculosis* but also nontuberculous mycobacteria (NTM) should be considered as possible pathogens. This possibility is of clinical importance because of the diagnostic challenges. Diagnosing NTM infections may require specific culture media and molecular assays. Under optimal conditions, cultures show growth of most NTM species (including *M*. *haemophilum*) within 2–3 weeks. NTM are less susceptible to antimicrobial drugs than *M. tuberculosis*. *M*. *haemophilum* is generally resistant in vitro to isoniazid, ethambutol, and rifampicin (Table), but no standardized susceptibility methods for *M*. *haemophilum* exist. Therefore, following current guidelines from the American Thoracic Society (ATS) is advisable for NTM infections (*9*).

No specific recommendations exist for pulmonary *M*. *haemophilum* infections, but for disseminated *M*. *haemophilum* infections, the ATS recommends a multidrug regimen combining clarithromycin, rifampicin/rifabutin, and ciprofloxacin. Although no studies on treatment duration for *M*. *haemophilum* infections have been conducted, the ATS guidelines recommend treatment until cultures taken during therapy are negative for 1 year (*9*). Whether TNF-αA treatment can be continued during antimycobacterial treatment is a matter of debate in the absence of sufficient safety data (*8*). In active tuberculosis infection, treatment with TNF-αA is contraindicated before patients complete a standard regimen of antituberculosis therapy; no information is available for NTM disease (*10*).

This case is presented especially to demonstrate the diagnostic challenges of NTM infections. For such cases, clinicians are advised to consult experts in the field of NTM infections.
